# Doxazosin Immediate Release as a Novel Treatment for Nightmares in Posttraumatic Stress Disorder

**DOI:** 10.1155/crps/6452923

**Published:** 2024-11-26

**Authors:** Danyaal Khan, Christie Richardson, Martin Forsberg

**Affiliations:** Department of Psychiatry, Rowan-Virtua School of Osteopathic Medicine, Stratford, New Jersey, USA

## Abstract

Nightmares and flashbacks are common debilitating symptoms of posttraumatic stress disorder (PTSD) that can disrupt daily functioning. Prazosin, an alpha-1 adrenergic antagonist, has been commonly used off-label for the treatment of these intrusion symptoms, although its short half-life makes it so that often multiple doses are needed. Doxazosin, another alpha-1 antagonist, is starting to be investigated in the treatment of PTSD-related nightmares due to its lesser side effect profile and longer half-life. In our case series, we present three cases of patients with PTSD-related nightmares who were successfully treated with doxazosin following a relapse of symptoms after discontinuation of prazosin for various reasons. The success of doxazosin immediate release for PTSD-related nightmares warrants further studies into its efficacy and use as an alternative treatment to prazosin.

## 1. Introduction

Posttraumatic stress disorder (PTSD) is a mental health disorder which is characterized by exposure to a traumatic event with subsequent avoidance, autonomic symptoms, intrusion symptoms (nightmares, flashbacks, etc.), and alterations in mood, cognition, arousal, and reactivity for at least 1 month [1]. PTSD may result after experiencing a life-threatening or horror-inducing event, such as exposure to war, combat, or sexual violence, and is a common diagnosis worldwide. The National Comorbidity Survey in 1995 studied a sample of 5,877 individuals aged 15–54 years in the United States and found an overall lifetime PTSD prevalence rate of 7.8% with rates for women at 10.4% and men at 5.0% [2]. Nightmares are a common symptom of PTSD, with studies showing a prevalence anywhere from 50% to 90% [3–5]. Flashbacks are common in PTSD as well (although less studied compared to nightmares), with a prevalence around 50%–80% [6, 7]. First-line treatments for PTSD involve psychological therapy and pharmacological interventions, which are commonly used in conjunction. Therapies include cognitive processing therapy, exposure therapy, trauma-focused therapy, psychodynamic therapies, cognitive behavioral therapy (CBT), and family therapy [8]. First-line pharmacological interventions mainly include selective serotonin reuptake inhibitors (SSRIs) such as fluoxetine, paroxetine, and sertraline, and selective serotonin and norepinephrine reuptake inhibitors (SNRIs) such as venlafaxine. Sertraline and paroxetine are the only medications approved by the US Food and Drug Administration (FDA) for the management of PTSD [9].

Prazosin, an alpha-1 adrenergic antagonist, which is FDA-approved for hypertension and benign prostatic hyperplasia, has historically been used off-label in PTSD for the treatment of nightmares, hyperarousal, and autonomic symptoms [10]. A recent systematic review pooling 10 research studies demonstrated the significant success of prazosin in reducing sleep disturbances in PTSD [11]. Prazosin's reported side effects are typically mild and transient in nature, which include dizziness, hypotension, headaches, palpitations, and syncope [12]. Recently, there have been case reports and studies using doxazosin, another alpha-1 adrenergic antagonist, in the treatment of PTSD. Doxazosin has been of interest due to its longer half-life of up to 22 h compared to prazosin's half-life of up to 3 h [13]. Prazosin typically needs to be dosed multiple times throughout the day to target daytime and nighttime symptoms of PTSD due to its short half-life, so once-daily dosing with doxazosin is thought to be more convenient and improve compliance [14]. A clinical trial of eight patients has found success in reducing symptoms of PTSD with doxazosin extended-release (XL) using PTSD rating scales [15]. Doxazosin has both immediate release (IR) and XL formulations, with the latter being used in the literature for PTSD and the focus of several studies [15–17]. Doxazosin XL starts at 4 mg dosing, and the capsule cannot be split if tolerability issues arise; however, doxazosin IR is available as a scored 1 mg tablet which allows for doses as low as 0.5 mg and slower titration, which could be especially useful in populations that are more sensitive to medications, such as the medically ill or geriatric patients. In this case series, we present three patients diagnosed with PTSD who were initially treated with prazosin with a reduction in trauma-related nightmares. In these reports, prazosin was discontinued for various reasons, and doxazosin IR was prescribed in place which had the same benefit with simpler dosing. This suggests that doxazosin IR has a long enough half-life to be dosed once a day to treat both daytime and nighttime symptoms of PTSD, which has not been documented in the literature that we know of. Although doxazosin XL is reported to be better tolerated than the IR formulation at similar dosing (8 mg), using the IR formulation may be especially useful for patients who are taking other blood pressure medications (and thus need a lower overall dose) or who experience adverse effects with the XL formulation (e.g., headaches, myalgia) [18]. We are not aware of any study evaluating a low dose of doxazosin IR for PTSD nightmares [19].

## 2. Case Presentations

### 2.1. Case 1

A 72-year-old male with a past psychiatric history of PTSD, obsessive compulsive disorder, and schizoaffective disorder, bipolar type, presented to our outpatient clinic with flashbacks, hypervigilance, nightmares, and insomnia. He had a history of childhood sexual and physical abuse, which manifested into persecutory and intrusive nightmares that greatly affected psychosocial functioning. At the time he was prescribed sertraline 200 mg daily, valproic acid 100 mg twice daily, aripiprazole 10 mg daily, and clonazepam 1 mg three times a day. He was also concurrently being treated with CBT. We started prazosin which was titrated to 2 mg three times a day, which led to, in the patient's words, “90% less nightmares.” He had already been on tamsulosin for benign prostatic hyperplasia, and his primary care provider preferred that prazosin be added rather than take the place of tamsulosin. Eventually, the patient experienced a complete remission of nightmares and flashbacks.

The patient remained on prazosin for 4 years but experienced a serious psychotic decompensation after aripiprazole was reduced. While hospitalized, prazosin was discontinued due to the concern for polypharmacy. Once discharged from the hospital and still psychotic, we restarted a lower prazosin dose of 1 mg twice daily to prevent the recurrence of PTSD symptoms. His psychosis went into remission once aripiprazole was increased and quetiapine was added. Eight months later, due to an increase in daytime PTSD symptoms, the patient was switched to doxazosin IR 1 mg daily. Other medications on doxazosin initiation included sertraline 250 mg daily, tamsulosin 0.4 mg daily, melatonin 5 mg nightly, quetiapine 50 mg daily/afternoon and 100 mg nightly, and aripiprazole 10 mg daily. Two months later, the patient described continued reduction in nightmares, no flashbacks, improved sleep, and denied dizziness. Doxazosin IR was titrated to 4 mg nightly, and after more than 2 years, the patient continued to report complete remission of nightmares and flashbacks (Table 1).

### 2.2. Case 2

A 73-year-old female presented to our outpatient clinic with a history of PTSD due to childhood physical and emotional abuse. She reported flashbacks, nightmares, sleep disturbances, and panic attacks. Her medication regimen on presentation included quetiapine 75 mg nightly, diazepam 10 mg four times a day, and doxepin 100 mg nightly. She was also treated concurrently with CBT. We started the patient on prazosin and titrated to 2 mg four times a day, which led to remission of PTSD symptoms, including nightmares. Four years after PTSD symptom remission, the patient decided to stop all her medications as she was feeling better, which led to hospitalization for status epilepticus, presumed to be due to the sudden discontinuation of benzodiazepines. Two months later, she reported a relapse of nightmares and anxiety, specifically that were reminiscent of prior abuse. She agreed to start doxazosin IR with the convenience of once-daily dosing, which was titrated to 8 mg nightly, and resulted in remission of nightmares. Due to some breakthrough daytime symptoms, doxazosin IR 2 mg daily was added, but she reported “blackout” and a fall (unclear if related to doxazosin, no blood pressure changes were noted), so the daytime dose was discontinued. She continued to have remission of nightmares for the following year (Table 1).

### 2.3. Case 3

A 63-year-old female presented to our outpatient clinic with a history of PTSD from physical and emotional abuse in adulthood. She reported a history of flashbacks and nightmares in remission on a psychotropic regimen of aripiprazole 20 mg daily, fluoxetine 20 mg daily, and prazosin 1 mg twice daily. She was also treated with CBT. About 5 years later, the patient discontinued all her psychotropic medications due to concern for sedation, although not clearly attributed to any particular medication. The patient continued remission of nightmares and flashbacks for the following 18 months until she experienced a trauma re-experiencing nightmare without being exposed to a trigger. She described that her nightmare was the same recurring nightmare she had before prazosin treatment. Due to daily dosing convenience, we started doxazosin IR 1 mg nightly which led to remission of nightmares for the next month (Table 1). The patient reported that she felt “good” and had no adverse effects.

## 3. Discussion

In our case series, three patients were initially treated with prazosin with remission of PTSD-related nightmares, but the medication was subsequently discontinued for various reasons. Each patient had a relapse of PTSD symptoms after stopping prazosin. Despite having tolerated and benefited from prazosin, doxazosin IR was initiated instead of restarting prazosin for the opportunity of once-daily dosing. Each patient benefited and tolerated the doxazosin IR. Case 1 patient had benefit with 3:2 of prazosin to doxazosin dose, Case 2 patient had a 1:1 ratio, and one patient had a 2:1 ratio. In our experience, typically patients do not require titration to a higher dose of doxazosin in comparison to prazosin, even when dosed once a day. Doxazosin was dosed at night as side effects such as hypotension are most likely to occur at peak plasma level (about 3 h after taking) and are less likely to be problematic if occurring overnight [19]. In general, patients should be educated about the risks of postural hypotension if getting up in the middle of the night and in the morning [19]. We also suggest that some patients (such as in Case 2) may require a higher dose of doxazosin, similar to prazosin, as studies have found some patients require up to 20 mg daily of prazosin for nightmare remission [17]. All patients were enrolled in trauma-focused CBT which can resolve the recurrence of symptoms on their own, but the timing of nightmare recurrence and remission after starting doxazosin had favorable correlation to the use of doxazosin. Overall, doxazosin IR was well-tolerated, with the exception of the patient in Case 2, who experienced a fall with an additional daily dose of doxazosin; however, hypotensive episodes were not recorded and could have been due to other medications or medical conditions. We present this series as a case for doxazosin to be utilized for PTSD symptoms, specifically nightmares, given its convenient once-daily dosing in comparison to prazosin.

According to the Veteran Affairs Administration (VA), CBT, or exposure therapy usually in combination with an SSRI [20] is recommended to be treated first-line for PTSD. Off-label treatments of PTSD may include atypical antipsychotics and alpha-1 adrenergic antagonists, such as prazosin, doxazosin, and terazosin [21, 22]. It is theorized that alpha-1 adrenergic antagonists reduce the burden of stress effects on the hypothalamic–pituitary–adrenal axis and norepinephrine surge during stressful events, which may reduce arousal symptoms in PTSD. Due to doxazosin being in the same class as prazosin, which has had off-label success in treating trauma-related nightmares, there is much interest in its efficacy given its longer half-life. Doxazosin differs from prazosin in that it has a lesser side effect profile and a longer half-life of 22 h versus 2–3 h for prazosin [23]. Doxazosin also has a better absorption profile, which minimizes the risk for hypotension [16]. Although doxazosin was initially thought to have limited blood–brain barrier penetration in comparison to prazosin, more recent studies have demonstrated doxazosin's ability to permeate the blood–brain barrier successfully [24].

Current research interest appears to be focusing on doxazosin XL due to the reduced risk of adverse effects compared to prazosin and doxazosin IR when used as an antihypertensive. In a review of a case report, open-label study, and retrospective review of doxazosin XL used for PTSD-related nightmares, doxazosin improved sleep quality and daytime functioning and decreased frequency and intensity of PTSD-related nightmares [16]. In a double-blind and placebo-controlled study by Rodgman et al. [15], eight participants with PTSD were treated with doxazosin XL, which was titrated to 16 mg daily, resulting in significant improvement in PTSD symptoms. Doxazosin IR has not been studied as extensively as doxazosin XL; however, we suggest that doxazosin IR should be considered for the treatment of trauma-related nightmares given its lower available initial dose, ability to be a split tablet if needed (in comparison to doxazosin XL which cannot be split), and generally lower cost at the pharmacy. The ability to start at a lower dose may be more beneficial in the geriatric and medically ill population who may already be on antihypertensives or experience postural hypotension with the XL formulation. In our case series, the patients were stable on doxazosin IR 1, 4, and 8 mg, showing the flexibility that can be used to achieve nightmare remission and the ability for slower titration.

The limitations of this case series were a small sample size, and patients were only seen by one psychiatrist. Another limitation is that it is difficult to isolate the exact nightmare remission in patients on multiple psychotropic regimens. However, this case series serves to provide important insight into the possible use of doxazosin IR as a treatment in trauma-related nightmares. Although not specifically analyzed in our cases, we theorize that doxazosin's longer half-life compared to prazosin can reduce trauma-related daytime arousal symptoms with once-daily dosing. Further studies and research on doxazosin IR used to treat trauma-related symptoms is recommended. Finally, although this series focuses on patients who have been diagnosed with PTSD, we recognize that many trauma patients may be suffering from related arousal symptoms without meeting the full Diagnostic and Statistical Manual of Mental Disorders-5 (DSM-5) criteria. Therefore, we suggest that future studies consider evaluating the utility of doxazosin IR in trauma survivors who may not meet the stringent PTSD diagnosis guidelines but are nonetheless suffering.

This case series evaluated the treatment of PTSD-related nightmares with doxazosin IR after discontinuation of prazosin. In our review of the literature, doxazosin IR has not been evaluated in the treatment of PTSD-related nightmares in favor of prazosin or doxazosin XL. Studies have been showing doxazosin XL can treat PTSD-related nightmares, but we suggest in our cases that doxazosin IR can also be just as efficacious in treating nightmares with a lower initial dose. We recommend that future studies consider doxazosin IR in the treatment of trauma-related nightmares and other PTSD arousal symptoms.

## Figures and Tables

**Table 1:** Treatment of prazosin versus doxazosin IR.

Case	Final dose of prazosin	Reported reason for prazosin discontinuation	Final dose of doxazosin IR	Time of treatment on doxazosin IR
Case 1	2 mg TID	To reduce polypharmacy	4 mg HS	26 months, 15 days
Case 2	2 mg QID	“Feeling better”	8 mg HS	20 months, 14 days
Case 3	1 mg BID	“Sedation”	1 mg HS	1 month, 5 days

Abbreviations: BID, two times a day; HS, nightly; QID, four times a day; TID, three times a day.

## Data Availability

The data that support the findings of this study are available on request from the corresponding author. The data are not publicly available due to privacy or ethical restrictions.
